# Contrast-Enhanced CT-Based Radiomics Analysis in Predicting Lymphovascular Invasion in Esophageal Squamous Cell Carcinoma

**DOI:** 10.3389/fonc.2021.644165

**Published:** 2021-05-14

**Authors:** Yang Li, Meng Yu, Guangda Wang, Li Yang, Chongfei Ma, Mingbo Wang, Meng Yue, Mengdi Cong, Jialiang Ren, Gaofeng Shi

**Affiliations:** ^1^ Department of Computed Tomography and Magnetic Resonance, Fourth Hospital of Hebei Medical University, Shijiazhuang, China; ^2^ Department of Cardiology, Second Hospital of Hebei Medical University, Shijiazhuang, China; ^3^ Department of Thoracic Surgery, Fourth Hospital of Hebei Medical University, Shijiazhuang, China; ^4^ Department of Pathology, Fourth Hospital of Hebei Medical University, Shijiazhuang, China; ^5^ Department of Computed Tomography and Magnetic Resonance, Children’s Hospital of Hebei Province, Shijiazhuang, China; ^6^ GE Healthcare China, Beijing, China

**Keywords:** lymphovascular invasion, radiomics, contrast-enhanced CT, nomogram, esophageal squamous cell carcinoma

## Abstract

**Objectives:**

To develop a radiomics model based on contrast-enhanced CT (CECT) to predict the lymphovascular invasion (LVI) in esophageal squamous cell carcinoma (ESCC) and provide decision-making support for clinicians.

**Patients and Methods:**

This retrospective study enrolled 334 patients with surgically resected and pathologically confirmed ESCC, including 96 patients with LVI and 238 patients without LVI. All enrolled patients were randomly divided into a training cohort and a testing cohort at a ratio of 7:3, with the training cohort containing 234 patients (68 patients with LVI and 166 without LVI) and the testing cohort containing 100 patients (28 patients with LVI and 72 without LVI). All patients underwent preoperative CECT scans within 2 weeks before operation. Quantitative radiomics features were extracted from CECT images, and the least absolute shrinkage and selection operator (LASSO) method was applied to select radiomics features. Logistic regression (Logistic), support vector machine (SVM), and decision tree (Tree) methods were separately used to establish radiomics models to predict the LVI status in ESCC, and the best model was selected to calculate Radscore, which combined with two clinical CT predictors to build a combined model. The clinical model was also developed by using logistic regression. The receiver characteristic curve (ROC) and decision curve (DCA) analysis were used to evaluate the model performance in predicting the LVI status in ESCC.

**Results:**

In the radiomics model, Sphericity and gray-level non-uniformity (GLNU) were the most significant radiomics features for predicting LVI. In the clinical model, the maximum tumor thickness based on CECT (cThick) in patients with LVI was significantly greater than that in patients without LVI (P<0.001). Patients with LVI had higher clinical N stage based on CECT (cN stage) than patients without LVI (*P*<0.001). The ROC analysis showed that both the radiomics model (AUC values were 0.847 and 0.826 in the training and testing cohort, respectively) and the combined model (0.876 and 0.867, respectively) performed better than the clinical model (0.775 and 0.798, respectively), with the combined model exhibiting the best performance.

**Conclusions:**

The combined model incorporating radiomics features and clinical CT predictors may potentially predict the LVI status in ESCC and provide support for clinical treatment decisions.

## Introduction

Esophageal cancer (EC) is the seventh most common cancer and the sixth most leading cause of cancer death worldwide, with an estimated 572,000 new cases and 509,000 deaths in 2018 (1). Esophageal squamous cell carcinoma (ESCC) is the primary histologic subtype of esophageal cancer, especially in high-incidence areas, such as China (2). Surgical resection of the tumor is the primary approach to treat esophageal cancer (3).

In recent years, despite improvements in staging, comprehensive treatment, and perioperative care, esophageal cancer remains a devastating disease, with a 5-year overall survival rate approximately ranging from 10–25% (4, 5). The main reasons for treatment failure are esophageal cancer recurrence and distant metastasis (5). Lymphovascular invasion (LVI) is a histopathological feature, usually defined as the presence of tumor cells within an endothelium-lined space, which is often referred to as lymph-vessel and blood-vessel (6, 7). The presence of LVI can only be identified if cancer cell clusters are found in the vascular-like endothelial lining structures (8, 9). LVI plays an important role in cancer cells spreading and lymph node metastasis, and it is associated with an increased risk of micrometastasis (10). Previous studies have reported that LVI is an indicator of poor prognosis in patients with esophageal cancer and is associated with early recurrence (6, 11).

In various situations for ESCC, LVI can serve as an indicator of highly aggressive behavior (12). Patients with LVI have a high risk of recurrence, so they must be treated with effective systemic therapy and intensive care (13). Therefore, identifying esophageal cancer with a high risk of recurrence, especially in patients with early recurrence, is crucial for an individualized treatment approach (3).

Currently, LVI can be diagnosed only by postoperative histopathology, and preoperative prediction is extremely difficult (14, 15). Compared with conventional CT, CECT can better distinguish normal tissues from tumors, and perform better in detecting tumors, showing tumor extent and staging (16, 17). Yin et al. (18) explored the correlation of triple-phase multi-slice CT scan with intratumor LVI of progressive gastric cancer. Ma et al. (14) found that multiphase dynamic CT could provide a non-invasive method for predicting LVI in gastric cancer through quantitative enhancement measurements. Conventional CT images are primarily used to extract morphological information from tumor tissues, but recent researches have shown that quantitative CT texture features can provide additional information (19, 20). Different from conventional CT image features, radiomics features can objectively reflect the heterogeneity of the tumor and allow more invisible information to be obtained (21, 22). Increasing studies have demonstrated the incremental value of texture analysis and radiomics approaches in predicting tumor grading, staging, response to treatment, and survival for gastrointestinal carcinoma (23–26). Through an in-depth analysis of image feature data, radiomics can quantitatively reveal predictive and prognostic associations between images and medical outcomes (27).

Recently, radiomics has been proven to be potential clinical value in predicting intra-tumoral LVI. Nie et al. (28) developed a radiomics nomogram incorporating Rad-score, clinical and PET/CT parameters to predict LVI in lung adenocarcinoma, which showed good predictive performance. Chen et al. (15) found that radiomics features based on CECT could serve as potential markers for predicting LVI and PFS in gastric cancer. The model established by radiomics features combined with clinical features has high diagnostic efficiency. Zhang et al. (29) revealed that multimodal radionics models based on MRI and CECT could be a useful tool for predicting LVI in rectal cancer.

Therefore, the aim of this retrospective study was to assess the feasibility of radiomics based on CECT to predict LVI in ESCC.

## Patients and Methods

### Patients

This retrospective study was performed following the Helsinki Declaration and approved by the Ethics Committee of our hospital to exempt patients from signing a written informed consent form. This study analyzed 726 patients with esophageal squamous cell carcinoma who underwent radical esophagectomy and confirmed by pathology in our hospital from August 2016 to October 2019. The inclusion criteria were as follows: 1) postoperative histopathology confirmed squamous cell carcinoma and the LVI status of the tumor tissue was explicit; 2) cases with completed clinicopathological data; 3) CECT performed before surgery within two weeks, with thin-section CECT images (1–2 mm) satisfying the diagnosis; 4) the region of interest could be measured on CECT images (tumor lesions larger than 5 mm); 5) no history of treatment for ESCC before operation. The exclusion criteria were as follows: 1) no precise pathological data or LVI status(n = 33); 2) other pathological types of esophageal cancer (n = 41); 3) no thin-section CECT images (n = 34); 4) any preoperative local or systemic treatment (n = 152); 5) no perceptible lesion on CECT images (n = 47); 6) poor image quality or noticeable artifacts affecting the assessment(n = 27); 7) with dual-source mode or gemstone spectral imaging mode (n = 58).

**Figure 1:**
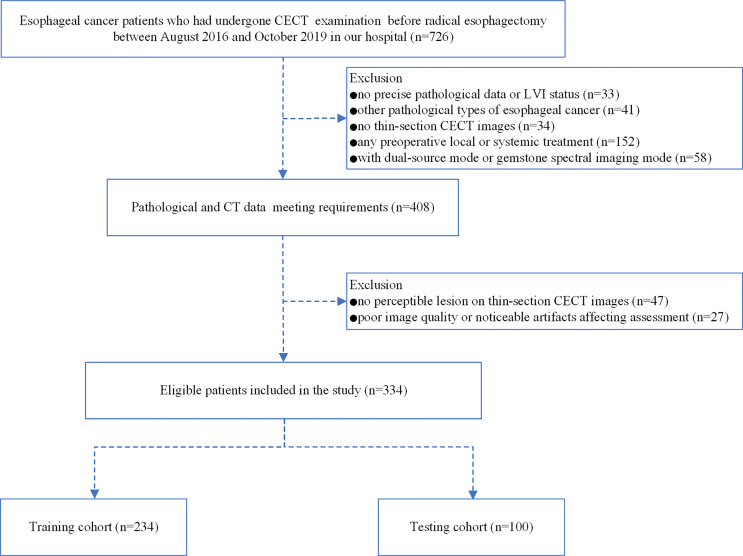
Flow chart illustrating the patient selection and exclusion criteria.

Finally, 334 patients were enrolled in the study. All enrolled patients were randomly divided into a training cohort and a testing cohort at a ratio of 7:3. **Figure 1** depicts the patient selection process.

### Clinical and Pathological Data

All enrolled patients were treated with surgical resection within two weeks after undergoing a CECT scan. Baseline clinicopathological data includes age, gender, carcinoembryonic antigen (CEA), squamous cell carcinoma antigen (SCCA), tumor differentiation, tumor infiltration depth, pathological T stage (pT stage), pathological N stage (pN stage), pathological AJCC stage (pAJCC stage), perineural invasion (PNI), and LVI status of the tumor.

The demographic information was retrieved from the HIS system. CEA and SCCA results were obtained by routine blood tests within two weeks before surgery. All histopathological parameters were obtained by analysis of all resected specimens by two pathologists. The pathological TNM stage was reclassified according to the American Joint Committee on Cancer (AJCC)/International Union Against Cancer International (UICC) 8th edition of the Cancer Staging Manual.

### CT Image Acquisition

All enrolled patients were requested to sign an informed consent form before undergoing a CECT examination. All patients underwent breathing training and were required to fast for 4 to 6 h prior to the CECT scan. To clean and dilate the esophageal and gastric lumen, patients were required to drink 500 to 1000 ml of purified water 1 to 5 min prior to the examination. No anticholinergic drugs were used in this study.

All CECT images were acquired on two commercial CT scanners. Scanner 1: a second-generation dual-source CT (SOMATOM Definition Flash, Siemens Healthcare, Forchheim, Germany) in the standard single-tube CT mode. The scanning parameters were as follows: tube voltage 120 kVp, automatic mA, slice thickness 5.0 mm, increment 5.0 mm, rotation time 0.5 s, pitch 1.2, reconstruction algorithm b20–40f, and reconstruction section thickness 1–2 mm. Scanner 2: a 256-slice CT (Revolution CT, GE Healthcare, Milwaukee, USA) in the standard single-energy CT mode. The scanning parameters were as follows: tube voltage 120 kVp, automatic mA, slice thickness 5.0 mm, increment 5.0 mm, rotation time 0.5 s, pitch 0.992:1, reconstruction algorithm standard, and reconstruction section thickness 1.25 mm.

All patients were in the supine position, and the scan covered the chest or chest plus abdomen. After intravenous injection of contrast agent (3.0–4.0 ml/s, 1.5 ml/kg, Iohexol,300 mg I/ml) via a syringe pump, an arterial phase scan was performed after a 30s delay, followed by a 20 ml saline flush.

The thin-section CECT images were exported from the PACS workstation in the DICOM format. The thin-section CECT images of each patient were imported into the Radiant software (V 4.6.9 https://www.radiantviewer.com/) for analysis separately. The tumor tissue appeared on CECT images as a thickened esophageal wall or a mass-like lesion with marked enhancement. The focal thickening of the esophageal wall of at least 5 mm or greater than the adjacent esophageal wall was identified as an abnormal thickening or tumor tissue (30). The thin-section CECT images were used for clinical TNM stage (31).

The maximum tumor thickness, as a potential predictive feature, was obtained by measuring on the maximum axial images. The measurement was performed using mediastinal window images (width, 400 HU; level, 40 HU), which can be adjusted appropriately for optimal display of the tumor tissue. The measuring and restaging procedure was performed by two radiologists with 10 years of experience in the diagnosis of esophageal cancer. When opinions differed in the measuring and restaging procedure, divergences were resolved by mutual consultation.

### Image Processing and Tumor Segmentation

The thin-section CECT images of each patient were uploaded to the open-source software 3D Slicer (version 4.10.2, https://www.slicer.org/). In order to eliminate the influence of different scanners, layer thicknesses and algorithms on the radiomic features, the following steps were carried out.

First, linear interpolation was adopted to 1 mm × 1 mm × 1 mm. Second, the images were discretized in grayscale with bandwidth set to 25, and the image filtering was processed applying Laplace of Gaussian (LoG, σ:3, 5, 7) and Wavelet (wavelet conversion, LLL, LLH, LHL, LHL, LHH, HLH, HHL, HHH) filter. The region of interest (ROI) was obtained by manually sketching layer by layer along the tumor edge to achieve segmentation. Considering the importance of tumor heterogeneity, the three-dimensional (3D) ROI encompassed the entire lesion, including internal areas of necrosis, but avoided including fatty tissues surrounding the lesion, lymph nodes, cardiac and lung tissues, blood vessels, bone tissues, intraluminal gas and fluid. After the sketching was finished, the ROI was modified with reference to the MPR images.

Radiologist 1 performed tumor segmentation on all 334 patients and radiologist 2 randomly selected 30 patients from the entire cohort for independent segmentation to assess inter-class agreement. Two weeks later, radiologist 1 repeated the independent segmentation of the previous 30 patients and evaluated the intra-class agreement with his own previous segmentation. Intra-and inter-class correlation coefficients (ICCs) was used to assess the intra-observer (radiologist 1 vs. radiologist 1) and inter-observer (radiologist 1 vs. radiologist 2) reproducibility of feature extraction.

### Radiomics Feature Extraction and Model Development

The radiomics feature extraction was performed using PyRadiomics software (32). A total of 1130 radiomics features were extracted including 18 classes of histogram features, 14 classes of shape factor feature, 24 classes of grayscale co-occurrence matrices (GLCM), 16 classes of grayscale travel matrices (GLRLM), 16 classes of grayscale region matrices (GLSZM), 14 classes of grayscale dependency matrices (GLDM), and five classes of adjacency domain matrices (NGTDM).

We performed three sequential steps for feature selection. First, we evaluated the inter-observer and intra-observer agreement of radiomic features and selected features with ICC values greater than 0.75 (15, 33–35). Second, Wilcoxon rank sum test (36, 37) was used to select features with *P* value less than 0.05. Third, the least absolute shrinkage and selection operator (LASSO) method was utilized to select the most useful predictive features in the training cohort. The lasso procedure is presented in **Figure S1** in the **Supplementary Material**.

Radiomics prediction models were developed based on three machine learning methods, namely logistic regression (Logistic), support vector machine (SVM) and decision tree (Tree), respectively. The best performing model was retained for adoption and radiomics score (Radscore) was then computed.

### Clinical Model Development

The clinical features analysis included gender, age, tumor location, CEA, SCCA, maximum tumor thickness based on CECT (cThick), clinical T stage base on CECT (cT stage), clinical N stage based on CECT (cN stage), and clinical AJCC stage based on CECT (cAJCC stage). The cT stage was performed according to the classification of CT staging standard suggested by Botet et al. (38) and Griffin Y et al. (30). The judgment of metastatic lymph nodes was based on the shortest diameter of enlarged lymph nodes in different regions (39), combined with lymph node axial ratio (40). The cN stage and cAJCC stage were restaged by the American Joint Committee on Cancer (AJCC)/Union Against Cancer International (UICC) eighth edition cancer staging manual.

First, univariate analysis of clinical features was performed to identify potential predictors associated with LVI. Second, multivariate analysis was performed with logistic regression, using statistically significant factors (*P* < 0.05) identified by univariate analysis, to screen out the independent predictive factors of LVI.

### Combined Model Development

**Figure 2:**
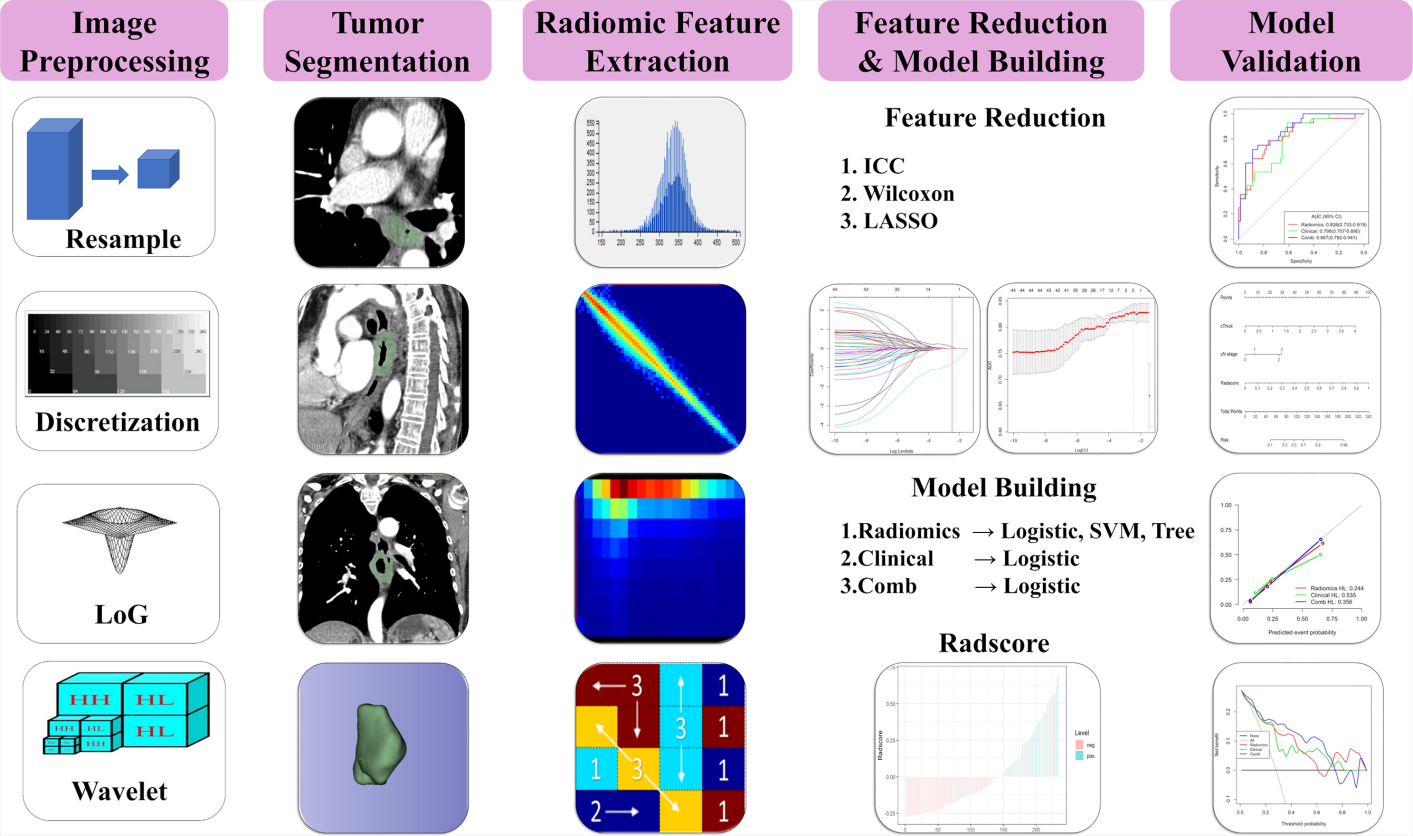
Radiomics prediction pipeline for LVI.

The independent predictive radiomics features generated from best performance machine learning model and the independent predictive clinical features were combined to develop a combined prediction model by logistic regression. Furthermore, a nomogram was also created in the training cohort and validated in the testing cohort. **Figure 2** illustrates the flowchart of the proposed analysis pipeline described above.

### Statistical Analysis

All statistical analysis was performed on R software (Version: 3.6.3, https://www.rproject.org/) in this study. The continuous variables were expressed as M±SD, and the categorical variables were reported as counts. For the analysis of clinical and pathological data, the Pearson’s Chi-squared test was used for categorical variables, and the Mann-Whitney U test was used for continuous variables with non-normal distribution. Trend test was used for ordinal variables. The reported statistical significance level was all two-sided, and the statistical significance level was set to 0.05.

The receiving operation characteristics (ROC) curves of each model were analyzed, and the area under the curve (AUC), accuracy, sensitivity, specificity, positive predictive value (PPV), and the negative predictive value (NPV) were calculated. The non-parametric Delong method was adopted to compare the statistical difference between AUC values. Calibration curves were plotted to determine the goodness-of-fit of the three models. The Hosmer-Lemeshow test was performed to test the reliability of calibration curves (41). Decision curve analysis (DCA) was used to calculate the clinical impact of the three models by quantifying the net benefit at different threshold probabilities.

## Results

### Patient Characteristics

Clinical and pathological data analysis of the 334 enrolled patients is summarized in **Table 1**. There were 96 patients (28.74%) with LVI and 238 patients (71.26%) without LVI. Patients with LVI had higher tumor differentiation, pT stage, pN stage, pAJCC stage, SCCA level, cN stage, cAJCC stage, and cThick than patients without LVI (P < 0.05). The differences in gender, age, tumor location, CEA level and cT stage between the two groups were not statistically significant (P > 0.05).

**Table 1:** Clinical and pathological characteristics of the patients.

Variables		LVI－ (n=238)	LVI+ (n=96)	Total (n=334)	*P*
**Gender**					0.652^1^
	**female**	83 (34.87)	31 (32.29)	114 (34.13)	
	**male**	155 (65.13)	65 (67.71)	220 (65.87)	
**Age**		63.18±7.10	62.64±7.55	63.02 ±7.23	0.457^2^
**pT stage**					0.038^3^
	**T1**	19 (7.98)	6 (6.25)	25 (7.48)	
	**T2**	56 (23.53)	11 (11.46)	67 (20.06)	
	**T3**	161 (67.65)	78 (81.25)	239 (71.56)	
	**T4**	2 (0.84)	1 (1.04)	3 (0.90)	
**pN stage**					<0.001^3^
	**N0**	142 (59.66)	20 (20.83)	162 (48.50)	
	**N1**	64 (26.89)	36 (37.50)	100 (29.94)	
	**N2**	25 (10.51)	24 (25.00)	49 (14.67)	
	**N3**	7 (2.94)	16 (16.67)	23 (6.89)	
**pAJCC stage**					<0.001^3^
	**I**	10 (4.21)	2 (2.08)	12 (3.59)	
	**II**	136 (57.14)	20 (20.83)	156 (46.71)	
	**III**	84 (35.29)	56 (58.34)	140 (41.92)	
	**IV**	8 (3.36)	18 (18.75)	26 (7.78)	
**Tumor differentiation**					0.009^3^
	**well**	2 (0.84)	0	2 (0.60)	
	**moderate**	174 (73.11)	55 (57.29)	229 (68.56)	
	**poor**	62 (26.05)	41 (42.71)	103 (30.84)	
**Tumor location**					0.071^1^
	**up**	20 (8.40)	2 (2.08)	22 (6.59)	
	**medium**	166 (69.75)	67 (69.79)	233 (69.76)	
	**low**	52 (21.85)	27 (28.13)	79 (23.65)	
**PNI**					0.027^1^
	**positive**	171 (71.85)	57 (59.37)	228 (68.26)	
	**negative**	67 (28.15)	39 (40.63)	106 (31.74)	
**CEA (ng/ml)**		2.95±1.41	2.99 ±1.24	2.96±1.36	0.959^2^
**SCCA (ng/ml)**		1.25±0.74	1.60 (1.62)	1.35±1.08	0.007^2^
**cT stage**					0.194^3^
	**T1**	0	2(2.08)	2(0.60)	
	**T2**	50(21.01)	10(10.42)	60(17.96)	
	**T3**	188(78.99)	84(87.50)	272(81.44)	
	**T4**	0	0	0	
**cN stage**					<0.001^3^
	**N0**	130 (54.62)	27 (28.13)	157 (47.01)	
	**N1**	90 (37.82)	33 (34.37)	123 (36.83)	
	**N2**	15 (6.30)	30 (31.25)	45 (13.47)	
	**N3**	3 (1.26)	6 (6.25)	9 (2.69)	
**cAJCC stage**					<0.001^3^
	**I**	0	0	0	
	**II**	140(58.82)	32(33.33)	172(51.50)	
	**III**	95(39.92)	62(64.59)	157(47.00)	
	**IV**	3(1.26)	2(2.08)	5(1.50)	
**cThick (cm)**		1.37 ±0.43	1.63 ±0.52	1.44±0.47	<0.001^2^
**Sphericity**		0.68±0.08	0.57±0.09	0.65±0.10	<0.001^2^
**GLNU**		58.81±42.91	99.54±95.20	70.52±65.10	<0.001^2^
**Radscore**		0.20±0.19	0.52±0.27	0.29±0.26	<0.001^2^
**Maximum3DDiameter(cm)**		4.21±1.54	5.78±1.97	4.66±18.19	<0.001^2^
**Mesh Volume (cm^3^)**		10.19±7.84	17.55±17.41	12.30±11.90	<0.001^2^

Unless otherwise indicated, data in parentheses are percentages. ^1^Pearson’s Chi-squared test; ^2^Mann-Whitney U test; ^3^Trend test for ordinal variables. LVI, lymphovascular invasion; pT stage, pathological T stage; pN stage, pathological N stage; pAJCC, pathological AJCC; cT stage, clinical T stage based on CECT; cN stage, clinical N stage based on CECT; cAJCC, clinical AJCC stage based on CECT; PNI, perineural invasion; CEA, Carcinoembryonic antigen; SCCA, Squamous Cell Carcinoma Antigen; cThick, maximum tumor thickness based on CECT; GLNU, Gray-Level Non-Uniformity.

### Radiomics Model Construction and Validation

To eliminate redundant features, highly correlated features with ICC values less than 0.75 would be excluded, with 233 features eliminated and 897 features retained. After screening out the redundant features by Wilcoxon analysis and LASSO, two most robust radiomics features (Sphericity and GLNU) were retained.

**Figure 3:**
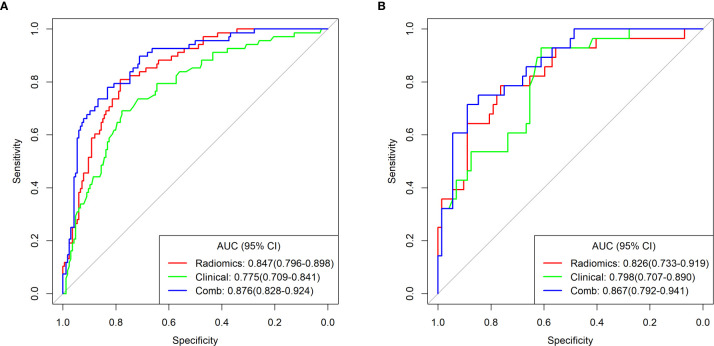
ROC curves of the radiomics, clinical and combined models for predicting LVI in the training cohort **(A)** and testing cohort **(B)**.

**Figure 4:**
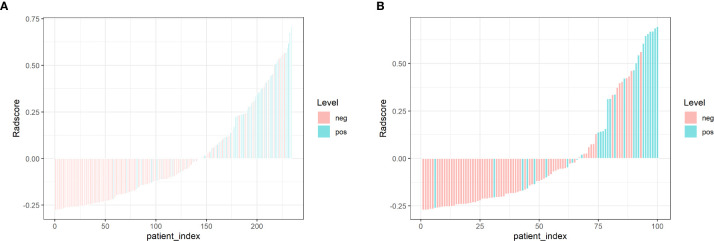
Bar charts of Radscore for each patient in the training cohort **(A)** and testing cohort **(B)**. The X-axis represents each patient, each bar represents one patient. Pink bars indicate the Radscore for patients without LVI, while light blue bars indicate the Radscore for patients with LVI. Pink bars above zero-line or light blue bars below the zero-line mean misclassification.

Logistic regression, SVM and Tree methods were separately used to establish the radiomics model. The model established by Logistic method yield the best performance, and the AUC values in the training and testing cohort were 0.847 and 0.826, respectively (**Table 3** and **Figure 3**). The Radscore for each patient was then calculated by a linear combination of the selected features weighted by their respective coefficients in the predictive model, which can be expressed as follows: Radscore = −1.2811–1.4584*Sphericity +0.4868* GLNU. Radscore for each patient in the training cohort and testing cohort is shown in **Figure 4**.

### Clinical Model Construction and Validation

Univariate analysis of clinical features revealed that cThick, cN stage, and SCCA level were significant association with LVI (**Table 2**). Multivariate analysis of significant variables revealed that cThick and cN stage were independent predictors of LVI (**Table 2**). The clinical prediction model, including the two clinical CT features, was established by logistic regression, with AUC values were 0.775 and 0.798 in the training and testing cohort, respectively (**Table 3** and **Figure 3**). Delong test shows that the AUC values of the clinical model were significantly lower than the AUC values of the radiomics model established by Logistic method in the training and testing cohort (P = 0.013, 0.030, **Table S1**).

**Table 2:** Univariate and Multivariate analysis to identify significant factors for LVI.

	Univariate		Multivariate	
	OR (95% CI)	*P*	OR (95% Cl)	*P*
**Gender**		0.747*	–	–
** female**	Reference		–	–
** male**	1.12(0.68-1.87)	0.658	–	–
**Age**	0.99(0.96-1.02)	0.548	–	–
**Location**		0.071*	–	–
** up**	Reference		–	–
** middle**	3.77(1.05-26.10)	0.040	–	–
** low**	4.83(1.26-34.60)	0.019	–	–
**CEA**	1.02(0.86-1.21)	0.811	–	–
**SCCA**	1.39(1.05-1.81)	0.043	–	–
**cT stage**	NA	NA	–	–
**cN stage**		<0.001*		<0.001*
** N0**	Reference		Reference	
** N1**	1.76(0.99-3.16)	0.054	2.58(1.27-5.37)	<0.001
** N2**	9.43(4.54-20.50)	<0.001	10.49(4.39-26.55)	<0.001
** N3**	9.22(2.21-48.70)	0.002	12.44(1.71-114.79)	0.014
**cAJCC**	NA	NA	–	–
**cThick**	3.30 (1.92-5.68)	<0.001	4.00(1.92-8.81)	<0.001

*Overall P value; OR, odds ratio; CI, confidence interval; cT stage, clinical T stage based on CECT; cN stage, clinical N stage based on CECT; cAJCC, clinical AJCC stage based on CECT; NA, not available. cThick, maximum tumor thickness based on CECT.

### Combined Model Construction and Validation

**Figure 5:**
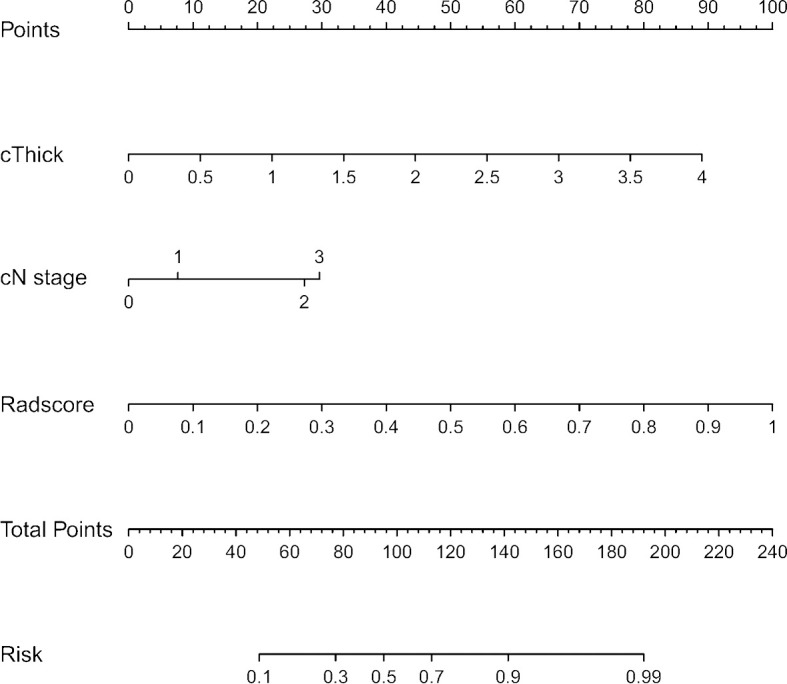
Nomogram for predicting LVI in ESCC. The nomogram was built in the training cohort with the independent predictors from radiomics model and clinical model.

**Figure 6:**
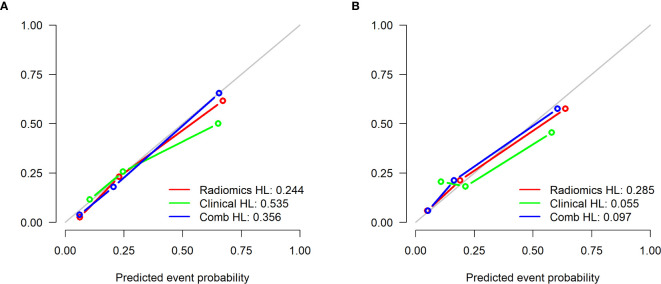
Calibration curves of the 3 models in the training cohort **(A)** and testing cohort **(B)**. The 45° gray line indicates perfect prediction and the colored lines the predictive performance of the different models. The closer the line fit to the ideal line, the better the predictive accuracy of the model.

Logistic regression was performed to establish a combined model incorporating the two radiomics independent predictors (Sphericity and GLNU) and two clinical independent predictors (cThick, cN stage), yielding AUC values of 0.876 and 0.867 in the training and testing cohort, respectively (**Table 3** and **Figure 3**). Based on this model in training cohort, a nomogram incorporated the four predictive factors was constructed to predict the individual probability of LVI (**Figure 5**). The Delong test revealed that the combined model and radiomics model were superior to the clinical model. In the training and testing cohort, calibration curves graphically showed good agreement between prediction and actual observation for the three models (**Figure 6**). The Hosmer-Lemeshow test yielded a nonsignificant statistic both in the training and testing cohort, which implied that there was no departure from perfect fit (training cohort: Radiomics 0.244, Clinical 0.535, Comb 0.356; testing cohort: Radiomics 0.285, Clinical 0.055, Comb 0.097).

**Table 3:** Diagnostic performance of individualized prediction models.

		AUC (95% CI)	ACC	SEN	SPE	PPV	NPV	Cutoff
**Training cohort (n=234)**								
** Radiomics**	**Logistic**	0.847(0.796-0.898)	0.791	0.809	0.783	0.604	0.909	0.287
	**Tree**	0.798(0.737-0.858)	0.786	0.765	0.795	0.605	0.892	0.210
	**SVM**	0.847(0.796-0.898)	0.791	0.809	0.783	0.604	0.909	0.282
** Clinical**		0.775(0.709-0.841)	0.752	0.691	0.777	0.560	0.860	0.309
** Comb**		0.876(0.828-0.924)	0.816	0.779	0.831	0.654	0.902	0.275
**Testing cohort (n=100)**								
** Radiomics**	**Logistic**	0.826(0.733-0.919)	0.760	0.679	0.792	0.559	0.864	0.284
	**Tree**	0.696(0.591-0.801)	0.730	0.643	0.764	0.514	0.846	0.200
	**SVM**	0.826(0.733-0.919)	0.760	0.679	0.792	0.559	0.864	0.281
** Clinical**		0.798(0.707-0.890)	0.650	0.607	0.667	0.415	0.814	0.300
** Comb**		0.867(0.792-0.941)	0.810	0.714	0.847	0.645	0.884	0.277

Logistic, logistic regression; Tree, decision tree; SVM, support vector machine; AUC, area under the curve; CI, confidence interval; ACC, Accuracy; SEN, Sensitivity; SPE, specificity; PPV, positive predictive value; NPV, negative predictive value. Radiomics, radiomics model; Clinical, clinical model; Comb, combined model.

**Figure 7:**
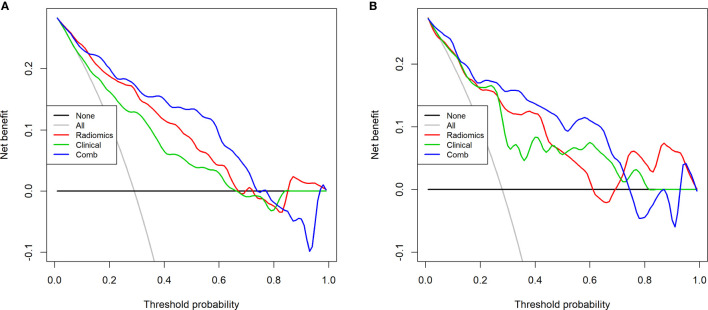
Decision curve analysis of the 3 models in the training cohort **(A)** and testing cohort **(B)**. The decision curve analysis (DCA) showed that the combined model yielded higher net benefit than the clinical model and the radiomics model, when the score is within a probability range from 0 to 0.720 in the training cohort and range from 0 to 0.728 in the testing cohort.

The decision curve analysis (DCA) showed that the combined model yielded a higher net benefit of LVI than the clinical model and the radiomics model within a probability range from 0 to 0.720 in the training cohort and range from 0 to 0.728 in the testing cohort (**Figure 7**). The decision curve analysis indicated that the combined model had better performance with higher overall benefits.

## Discussion

As a routine examination, CECT is a useful tool for differential diagnosis, preoperative evaluation, treatment, and prognosis of patients with esophageal cancer (30, 31, 42–45). The significance of the present study is that it proposes a novel method for predicting LVI in ESCC for the first time. It can be concluded that Radscore, a quantitative parameter based on CECT radiomics feature, could serve as an independent predictor of LVI in ESCC, and that the radiomics model combined with clinical features based on CECT can improve the predictive ability. This novel approach is expected to provide risk stratification and support decision-making in clinical treatment for patients with ESCC.

Currently, the AJCC/UICC guidelines have not incorporated LVI as an independent prognostic indicator for esophageal cancer in the TNM staging system. Pathological studies have now incorporated LVI into the TNM staging system for multiple cancers (46, 47). Many studies have revealed that LVI is an independent risk factor for survival in patients with ESCC (44, 48). Preoperative prediction of LVI status is necessary for patients to implement an aggressive treatment plan (49). Patients with suspected tumor microvascular invasion require more advanced treatment, such as more extensive surgery or preoperative adjuvant therapy (50).

In the clinical model we established, the univariable analysis identified that cThick, cN stage, and SCCA level were associated with LVI. According to multivariate analysis, cThick and cN stage were independent predictors of LVI. The maximum tumor thickness reflects the tumor infiltration depth, which correlates with the development of LVI (51). The incidence of LVI increases with the tumor infiltration depth (8, 52). On CECT images, identification tumor region usually depended on the extent of invasion by the thickness of esophageal wall, and it was generally considered that thickness > 5 mm was abnormal. The CECT has exhibited significant advantages in measuring tumor thickness (43), which allows for initial preoperative T staging. The multivariate analysis showed that cThick was an independent predictor of LVI. This indicated that the cThick could reflect the degree of tumor invasion more robustly and thus better predict the status of LVI than the cT stage. In the clinical model, cN stage was another independent predictor of LVI. In general, CT has low sensitivity in detecting metastases according to conventional criteria (53). New diagnostic criterion for MDCT improves the sensitivity of detection of lymphatic metastasis (40), so that the utilization of CECT for cN stage is more consistent with the clinical practice. The clinicopathological data revealed that patients with LVI had higher pN stage than patients without LVI, which was consistent with the cN stage results. Clinically, LVI may be an upgrade factor for all N stages (54), particularly in patients with negative lymph node metastases, for it is the only factor that affects the prognosis (55). In our study, the radiomics model achieved AUCs of 0.847 and 0.826 in the training and testing cohort, which were better than the AUCs of the clinical model (0.775 and 0.798, respectively).

In a prior study, Chen et al. (15) used arterial-venous phase CECT images to build radiomics models to predict the LVI status in gastric cancer. The results showed that the combined model based on arterial-venous phase radiomics combining with clinical risk factors had the best performance with AUC values of 0.856 and 0.792 in the training and test groups. The performance of this combined model was similar to ours. But the difference lied in that our radiomics model was based on the single arterial phase CECT images and did not include postoperative pathological factors. However, for esophageal cancer, plain and venous phase CECT scans were not the routine sequences, while a single arterial phase is more in line with clinical practice. Zhang et al. (29) established multimodal imaging radiomics model using MRI (T2WI, DWI) and venous phase CECT images to predict LVI status in rectal cancer, yielding the best performance compared with every single model. This implies that incorporating MRI or PET/CT images into our model to develop a multimodal radiomics model may improve the predictive performance. Nie et al. (28) found that the prediction model developed using CT morphology, 2D-RS and SUV values (AUCs,0.851 and 0.838, in training and testing cohort) performed better than the model without SUV values (0.796,0.822), reflecting the incremental value of metabolic parameters in the prediction of LVI in LAC patients. The difference from our study was that the authors adopted 2D-ROI (CT) for radiomics feature extraction and model building. As for esophageal cancer, the tumor tissue has a variable length. The selection of largest cross-sectional area is elusive and is hard to achieve agreements among different performers. Theoretically, 3D-ROI(CT) which we adopted can better reflect the heterogeneity of the whole tumor than 2D-ROI. However, our study did not compare the performance of the two prediction models built on 2D-ROI and 3D-ROI.

However, incorporating radiomics into predictive studies requires a multi-step process that includes reliable statistical analyses such as feature selection and classification to reduce over-fitting and to build robust predictive or prognostic models (56). Although several machine learning methods (alone or in combination) have been used in radiomics analysis for feature selection and classification, there is no “one-size-fits-all” approach since the performance of the workflow of various machine learning methods is application and/or data type dependent (57). Isaac et al. (57) provided a cross-sectional combination of 6 feature choices and 12 classifiers for multimodal imaging radiomics-based prediction of EGFR and KRAS mutation status in NSCLC patients, and the results showed that different combinations of features, classifiers and image settings had different diagnostic performance (AUCs ranged from 0.5 to 0.82). Similarly, Rastegar et al. (58) compared 4 feature selection methods and 4 classification methods, founding that different combinations of screening methods with different classifiers had different and variable performance in predicting bone mineral loss at different sites. In another previous study, Ghasem et al. (59) compared seven different feature selection methods and 12 classifiers, in which heatmaps were adopted to show their cross-combinations. However, our study did not analyze so many different feature extraction methods and classification methods, as well as their combinations. In the model building process, we selected only three machine learning algorithms, namely Logistic, SVM and Tree, to select the best radiomics model. Our results showed that the radiomics model built by Logistic method was the best, and the difference between Logistic method and SVM method was not statistically significant, but the difference between Logistic method and Tree method was statistically significant (**Table S1** in **Supplementary Material**). Furthermore, whether filter models or classifiers have a greater impact on model performance has been inconsistently reported in various studies. Parmar et al. (60) evaluated the performance and stability of 13 feature selection methods and 11 machine learning classification methods in predicting overall survival of patients with head and neck cancer. They concluded that the classification method had the greatest impact on performance and should be chosen with careful consideration. Stefan Leger et al. (61) assessed the performance of 11 machine learning algorithms combined with 12 feature selection methods by the concordance index (C-Index), to predict loco-regional tumor control (LRC) and overall survival for patients with head and neck squamous cell carcinoma. They reported that the performance differences between the learning algorithms were smaller than the differences between the feature selection methods. In summary, determining the appropriate feature selection method and learning algorithm is a key step in building an accurate radiomics model, which needs to be compared and selected according to the specific type of study.

In our radiomics model, among 1130 radiomics features, Sphericity and GLNU were the most significant components for predicting histological LVI status. The detailed descriptions and equations of all relevant radiomics features are presented in **Table S2** in the **Supplementary Material**. Sphericity is a radiomics shape feature that describes how close a given volume is to a perfect sphere (62). The value range is 0 < Sphericity ≤ 1, where a value of 1 indicates a perfect sphere (63). As a dimensionless measure, Sphericity is independent of scale and orientation. Compared with other radiomics features, Sphericity is characterized by high reproducibility (64). The Sphericity is independent of the segmentation method but related to the corresponding tumor volume, while larger volumes exhibit lower Sphericity (65). The Sphericity should be prioritized as these have minimal variations with volume changes, slice thickness and resampling (63). Perhaps due to our adoption of two types of CT scanners with different thickness and reconstruction algorithms, Sphericity was retained as a robust radiomics feature. Clinically, Sphericity can predict tumor grade, local failure, and OS in patients with meningioma, and low Sphericity is a predictor of poor preoperative imaging outcome (66). As for breast cancer, Sphericity can predict the expression of Ki-67, which correlates with the malignancy of the tumor (67). Sphericity also can serve as an noninvasive imaging biomarker to identify cancer subtype (68–70) and predict the pathological response (71). Our study showed that tumors with LVI had lower Sphericity values than tumors without LVI, indicating that tumors with low Sphericity were more likely to develop LVI. This also explored the high invasiveness of tumors with LVI from another aspect.

In our study, GLNU was another independent predictor for LVI. Gray-level non-uniformity (GLNU) is a measure of the similarity of gray-level values throughout the image (72). Many radiomics features are unstable in different reconstruction algorithms, while GLNU is one of the most repetitive radiomics features showing good stability (73). The GLNU is less sensitive to reconstructed convolutional kernels and thus has higher stability under different image reconstruction algorithms (74). However, GLNU is sensitive to both voxel size and number of gray levels, therefore, it requires normalization by voxel size and number of gray levels (75). The GLNU increases with the tumor heterogeneity, which is related to tumor invasion, treatment response and prognosis (76). As an independent risk factor for poor prognosis, high GLNU is associated with worse survival in patients with pancreatic cancer who have undergone surgery (72). Our study showed that tumors with LVI had higher GLNU values than those without LVI, while the presence of LVI implies an increase in tumor heterogeneity. The GLNU can be used precisely as a predictor of LVI, reflecting the heterogeneity and aggressiveness. This finding was consistent with the results of previous studies of renal cell carcinoma, which indicated that higher GLNU values had greater heterogeneity and invasiveness (76).

In addition, two additional radiomics features were specifically extracted, namely the maximum 3D diameter and the Mesh Volume (**Table 1**), even though the two radiomics features were not independent predictors. The result showed that patients with LVI-positive had greater maximum 3D diameter and Mesh Volume than patients without LVI (p < 0.001), which was consistent with previous studies on the prediction of LVI in gastric and hepatocellular carcinoma (15, 25). Since there was no reliable individual factor to predict LVI, a predictive model combining radiomics and clinical features would be viable. By incorporating cThick and cN stage into the radiomics model, the AUCs of the combined model in the training and testing cohort were improved to 0.876 and 0.867, respectively.

However, our study had several limitations. Firstly, this was a single-center retrospective study, and the enrolled patients included only those who had undergone surgery, which may introduce a selection bias. Secondly, the sample size was relatively small, and the resulting sample error causes the performance of the prediction model in the testing cohort to be slightly lower than that in the training cohort. Thirdly, as this study was a retrospective study without plain and venous phase scanning, more meaningful qualitative and quantitative parameters were not included. Fourthly, we did not evaluate the robustness of the radiomics features between the two CT scanners. Finally, this study did not evaluate the value of radiomics based on CECT in predicting the prognosis of ESCC patients with LVI, which may be the next step in our research.

## Conclusion

The radiomics features based on CECT can serve as potential indicators to predict LVI in ESCC. The combined model incorporating both radiomics and clinical features yielded better predictive performance for LVI in ESCC. Considering that it is a single-center study based on arterial phase CECT images, future validation studies with multiple phases and multiple centers are needed to verify its clinical feasibility.

## Data Availability Statement

The datasets for this manuscript are not publicly available because involving patient confidential information. Requests to access the datasets should be directed to the corresponding author.

## Ethics Statement

The studies involving human participants were reviewed and approved by ethics committee of the Fourth Hospital of Hebei Medical University. Written informed consent for participation was not required for this study in accordance with the national legislation and the institutional requirements.

## Author Contributions

YL and JR designed the study. YL and MYu wrote the initial draft of the manuscript and accomplished the final version. GW and YL collected the required CT data. MW and MYue provided and analyzed the surgical and pathological data required for the study. MC collected and assembled the total data. JR performed the statistical analysis and interpretation. YL determined the selection of references and experimental standards. YL and CM performed the CT data analysis and interpretation. GS performed manuscript approval and modification. All authors contributed to the article and approved the submitted version.

## Funding

This study was supported by the Medical Science Research Project Plan of Hebei Province (Grant No. 20210631).

## Conflict of Interest

JR was employed by GE Healthcare China.

The remaining authors declare that the research was conducted in the absence of any commercial or financial relationships that could be construed as a potential conflict of interest.
