# Corrigendum: Contrast-Enhanced CT-Based Radiomics Analysis in Predicting Lymphovascular Invasion in Esophageal Squamous Cell Carcinoma

**DOI:** 10.3389/fonc.2021.712493

**Published:** 2021-06-17

**Authors:** Yang Li, Meng Yu, Guangda Wang, Li Yang, Chongfei Ma, Mingbo Wang, Meng Yue, Mengdi Cong, Jialiang Ren, Gaofeng Shi

**Affiliations:** ^1^ Department of Computed Tomography and Magnetic Resonance, Fourth Hospital of Hebei Medical University, Shijiazhuang, China; ^2^ Department of Cardiology, Second Hospital of Hebei Medical University, Shijiazhuang, China; ^3^ Department of Thoracic Surgery, Fourth Hospital of Hebei Medical University, Shijiazhuang, China; ^4^ Department of Pathology, Fourth Hospital of Hebei Medical University, Shijiazhuang, China; ^5^ Department of Computed Tomography and Magnetic Resonance, Children’s Hospital of Hebei Province, Shijiazhuang, China; ^6^ GE Healthcare China, Beijing, China

**Keywords:** lymphovascular invasion, radiomics, contrast-enhanced CT, nomogram, esophageal squamous cell carcinoma

In the original article, there was a mistake in **Table 2** as published.

During the analysis using the statistical software, a display problem caused the OR value to be incorrectly displayed as 1/OR. The same error occurred for the 95% CI. This was only a display error and did not affect the other tables, figures and final results.

The corrected **Table 2** appears below.

**Table 2:** Univariate and Multivariate analysis to identify significant factors for LVI.

	Univariate		Multivariate	
	OR (95% CI)	*P*	OR (95% Cl)	*P*
**Gender**		0.747*	–	–
** female**	Reference		–	–
** male**	1.12(0.68-1.87)	0.658	–	–
**Age**	0.99(0.96-1.02)	0.548	–	–
**Location**		0.071*	–	–
** up**	Reference		–	–
** middle**	3.77(1.05-26.10)	0.040	–	–
** low**	4.83(1.26-34.60)	0.019	–	–
**CEA**	1.02(0.86-1.21)	0.811	–	–
**SCCA**	1.39(1.05-1.81)	0.043	–	–
**cT stage**	NA	NA	–	–
**cN stage**		<0.001*		<0.001*
** N0**	Reference		Reference	
** N1**	1.76(0.99-3.16)	0.054	2.58(1.27-5.37)	<0.001
** N2**	9.43(4.54-20.50)	<0.001	10.49(4.39-26.55)	<0.001
** N3**	9.22(2.21-48.70)	0.002	12.44(1.71-114.79)	0.014
**cAJCC**	NA	NA	–	–
**cThick**	3.30 (1.92-5.68)	<0.001	4.00(1.92-8.81)	<0.001

*Overall P value; OR, odds ratio; CI, confidence interval; cT stage, clinical T stage based on CECT; cN stage, clinical N stage based on CECT; cAJCC, clinical AJCC stage based on CECT; NA, not available. cThick, maximum tumor thickness based on CECT.

The authors apologize for this error and state that this does not change the scientific conclusions of the article in any way. The original article has been updated.

