# Supplementary material for: Contrast-Enhanced CT-Based Radiomics Analysis in Predicting Lymphovascular Invasion in Esophageal Squamous Cell Carcinoma
Source: Front Oncol. 2021 May 14;11:644165. doi: 10.3389/fonc.2021.644165 (PMC8162215; doi:10.3389/fonc.2021.644165)
Supplement: Supplementary file 1 [file DataSheet_1.docx]

Contrast-enhanced CT Based Radiomics Analysis in Predicting Lymphovascular Invasion in E[sophageal Squamous Cell Carcinoma](javascript:showjdsw('showjd_0','j_0'))

A B


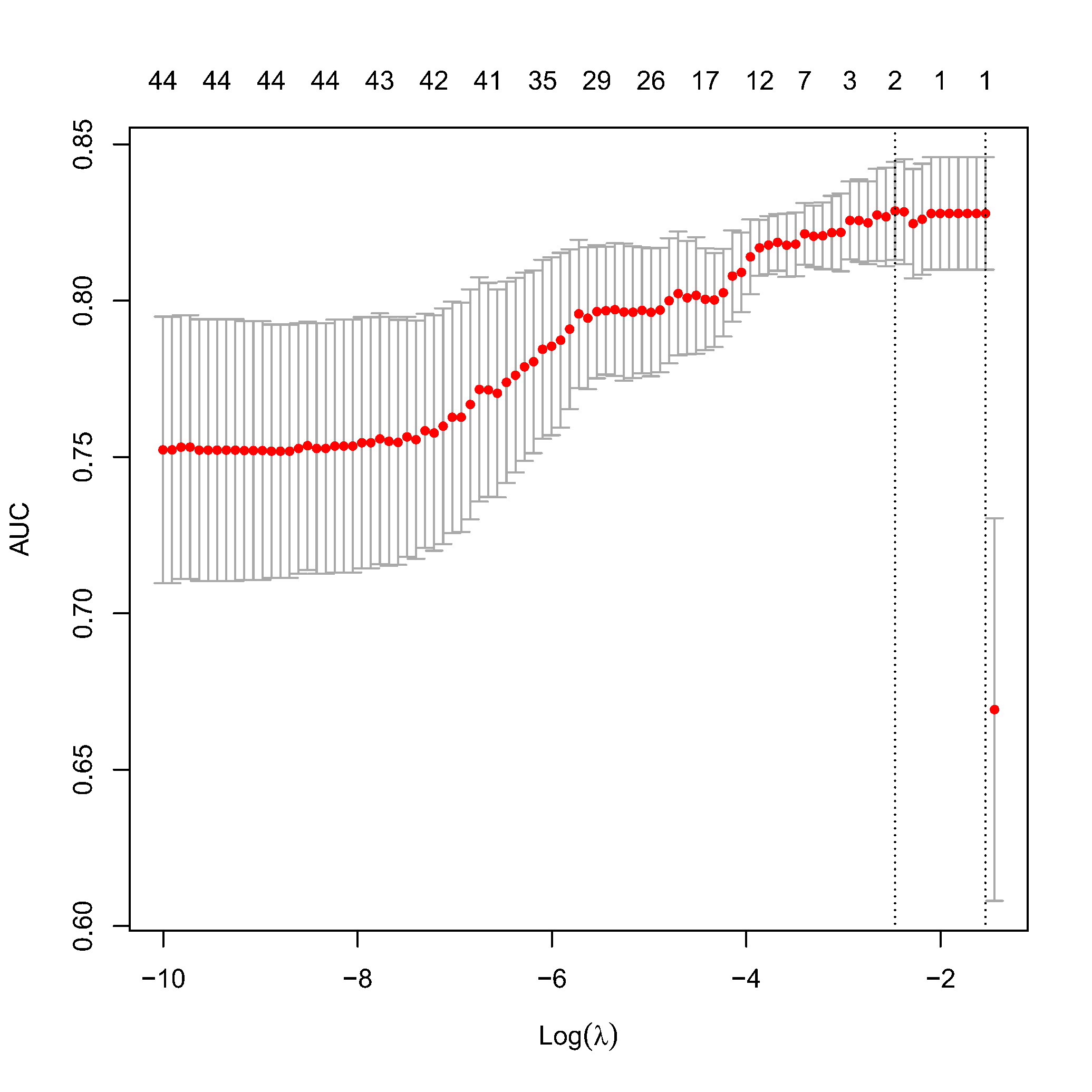

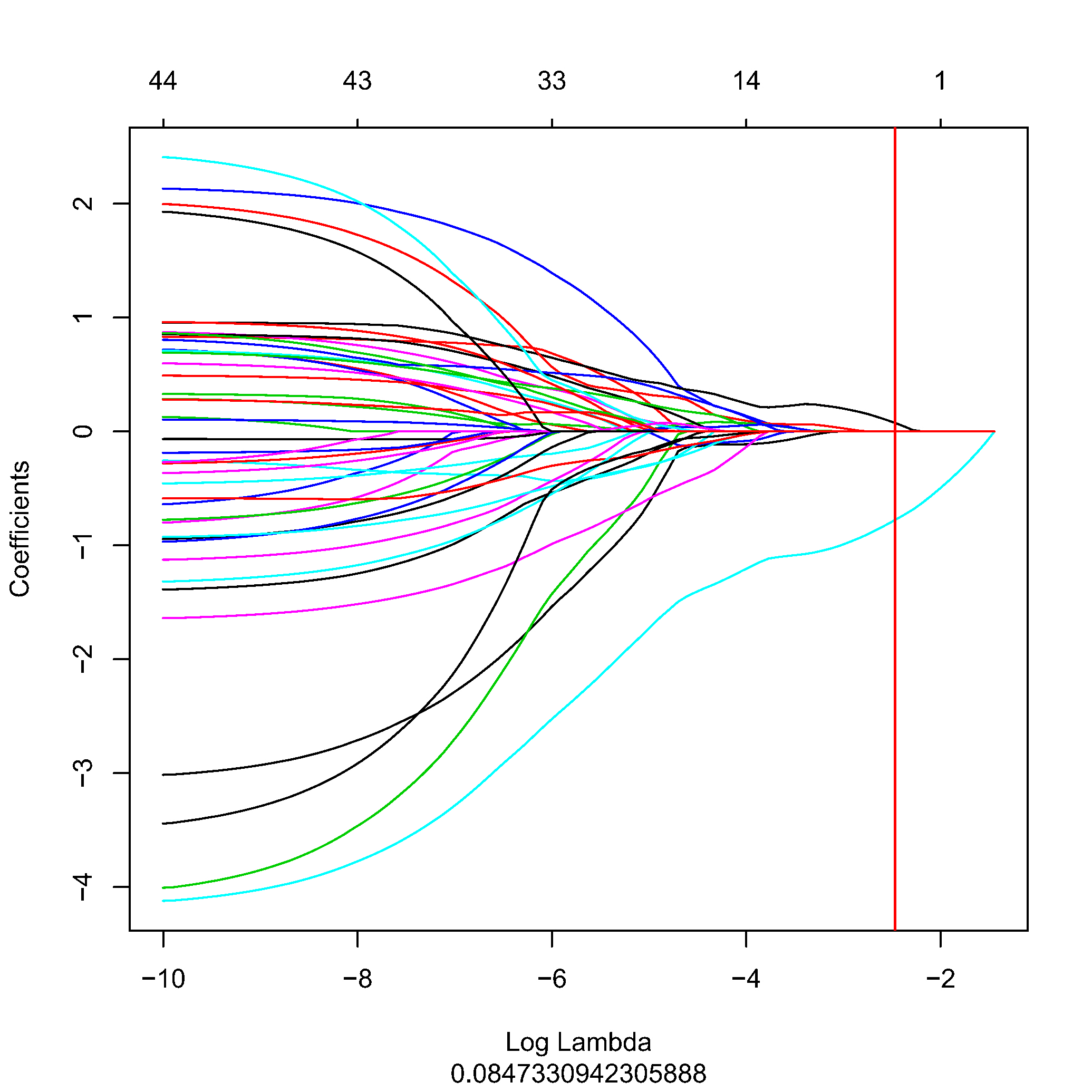


**Figure S1**. The least absolute shrinkage and selection operator (LASSO) binary logistic regression model used to select texture feature. (A) Tuning parameter (λ) selection in the LASSO model used 10-fold cross-validation via minimum criteria. The area under the receiver operating characteristic curve (AUC) is plotted versus log(λ). Dotted vertical lines are drawn at the optimal values by using the maximum AUC value. log(λ)= 0.0847, with λ chosen of -2.468. (B) LASSO coefficient profiles of the 2 texture features. A coefficient profile plot is produced against the log(λ) sequence. Vertical line is drawn at the value selected using 10-fold cross validation, where optimal λ results in 2 non-zero coefficients.

**Table S1. Delong test for the different models**

|  |  | **Training cohort** | **Testing cohort** |
| --- | --- | --- | --- |
|  |  | *P* value | *P* value |
| Algorithms | Logistic-Tree | 0.008214876 | 0.00068 |
|  | Logistic-SVM | 0.918501019 | 1 |
|  | Tree-SVM | 0.006566503 | 0.000504 |
| Models | Radiomics-Clinical | 0.013305854 | 0.029964 |
|  | Radiomics-Comb | 0.230774756 | 0.940094 |
|  | Clinical-Comb | 0.000137463 | 0.010272 |

Logistic, logistic regression; SVM, support vector machine; Tree, decision tree; radiomics, radiomics model; Clinical, clinical model; Comb, combined model.

**Table S2. Description and equation of the radiomics features**

| **Index** | **Feature name** | **Description** | **Equation** |
| --- | --- | --- | --- |
| **1** | **Sphericity** | Sphericity is a measure of the roundness of the shape of the tumor region relative to a sphere. It is a dimensionless measure, independent of scale and orientation. The value range is 0<sphericity≤1, where a value of 1 indicates a perfect sphere (a sphere has the smallest possible surface area for a given volume, compared to other solids) | $\frac{\left( \pi\right)^{1/3}\left( 6V \right)^{\frac{2}{3}}}{A}$. |
| **2** | **GrayLevelNonUniformity (GLNU)** | The variability of gray-level intensity values in the image, with a lower value indicating more homogeneity in intensity values | $\frac{\sum_{i=1}^{N_{g}} \left( \sum_{j=1}^{N_{d}} \boldsymbol{P}(i,j) \right)^{2}}{N_{z}}$ |
| **3** | **Maximum3DDiameter** | Maximum 3D diameter is defined as the largest pairwise Euclidean distance between tumor surface mesh vertices. Also known as Feret Diameter. | / |
| **4** | **Mesh Volume** | The volume of the ROI V is calculated from the triangle mesh of the ROI. For each face *i* in the mesh, defined by points *a_i_*, *b_i_* and *c_i_*, the (signed) volume V*_f_* of the tetrahedron defined by that face and the origin of the image (*O*) is calculated. (1) The sign of the volume is determined by the sign of the normal, which must be consistently defined as either facing outward or inward of the ROI. Then taking the sum of all *V_i_*, the total volume of the ROI is obtained (2). | $V_{i}$=$\frac{O_{a_{i}}\cdot\left( O_{b_{i}}\times O_{c_{i}} \right)}{6}$ (1)  *V*=$\sum_{i=1}^{N_{f}} V_{i}$ (2) |
